# Interaction of the Nitrogen Regulatory Protein GlnB (P_II_) with Biotin Carboxyl Carrier Protein (BCCP) Controls Acetyl-CoA Levels in the Cyanobacterium *Synechocystis* sp. PCC 6803

**DOI:** 10.3389/fmicb.2016.01700

**Published:** 2016-10-26

**Authors:** Waldemar Hauf, Katharina Schmid, Edileusa C. M. Gerhardt, Luciano F. Huergo, Karl Forchhammer

**Affiliations:** ^1^Interfaculty Institute of Microbiology and Infection Medicine Tübingen, Eberhard-Karls-Universität TübingenTübingen, Germany; ^2^Departamento de Bioquímica e Biologia Molecular, Universidade Federal do ParanáCuritiba, Brazil; ^3^Setor Litoral, Universidade Federal do ParanáMatinhos, Brazil

**Keywords:** acetyl-CoA, GlnB (P_II_), BCCP, cyanobacteria, *Synechocystis* sp. PCC 6803

## Abstract

The family of P_II_ signal transduction proteins (members GlnB, GlnK, NifI) plays key roles in various cellular processes related to nitrogen metabolism at different functional levels. Recent studies implied that P_II_ proteins may also be involved in the regulation of fatty acid metabolism, since GlnB proteins from Proteobacteria and from *Arabidopsis thaliana* were shown to interact with biotin carboxyl carrier protein (BCCP) of acetyl-CoA carboxylase (ACC). In case of *Escherichia coli* ACCase, this interaction reduces the k_cat_ of acetyl-CoA carboxylation, which should have a marked impact on the acetyl-CoA metabolism. In this study we show that the P_II_ protein of a unicellular cyanobacterium inhibits the biosynthetic activity of *E. coli* ACC and also interacts with cyanobacterial BCCP in an ATP and 2-oxoglutarate dependent manner. In a P_II_ mutant strain of *Synechocystis* strain PCC 6803, the lacking control leads to reduced acetyl-CoA levels, slightly increased levels of fatty acids and formation of lipid bodies as well as an altered fatty acid composition.

## Introduction

*De novo* fatty acid biosynthesis is an essential metabolic step for microbial growth as it provides fatty acids for phospholipid biosynthesis, which is crucial for the integrity of the cell membrane. The first and committed step in fatty acid biosynthesis is catalyzed by the enzyme acetyl-CoA carboxylase (ACC). In bacteria, the ACC enzyme complex consists of three functional units: i) the biotin carboxyl carrier protein (BCCP, *accB*) is covalently modified at a conserved lysine residue with biotin; ii) biotin carboxylase (BC, *accC*) carboxylates the biotin residue during the catalytic cycle in an ATP-dependent manner and iii) carboxyl transferase (CT, *accA* and *accD*) translocates the “activated” CO_2_ in the active site from biotin to acetyl-CoA forming malonyl-CoA, the substrate for fatty acid elongation (Cronan and Waldrop, 2002). Biosynthetic activity of ACC is subjected to tight regulation by several mechanisms. The enzyme is feedback inhibited by acyl-ACP (Jiang and Cronan, 1994) and the catalytic activity of CT is decreased by its own transcript when acetyl-CoA levels are low. Evidence that the CT component additionally represses the translation of the *accA/accD* mRNA (Meades et al., 2010) has been challenged lately (Smith and Cronan, 2014). Disturbance of ACC regulation has been shown to impact the acetyl-CoA pool (Davis et al., 2000; Zha et al., 2009). Recent findings showed that ACC in *Arabidopsis thaliana* and *Escherichia coli* is regulated additionally through interaction of BCCP with the P_II_ protein GlnB (Feria Bourrellier et al., 2010; Gerhardt et al., 2015).

P_II_ proteins are small homotrimeric signal transduction proteins with binding sites for ATP/ADP and 2-OG at the three intersubunit-clefts and large flexible T-loops emanating from these sites, with the T-loop conformation reflecting the ligand binding status (Forchhammer and Lüddecke, 2016). Furthermore, the T-loops may be covalently modified in their apical region, either by uridylylation or adenylylation at Tyr51 or by phosphorylation of Ser49 in cyanobacteria (Leigh and Dodsworth, 2007; Merrick, 2014; Forchhammer and Lüddecke, 2016). In most cases, covalent modification negatively affects interaction of P_II_ with its targets. In unicellular cyanobacteria, two P_II_ partners have been characterized; the transcriptional co-activator PipX and the key enzyme of arginine synthesis, N-acetyl glutamate kinase (NAGK). PipX interaction with P_II_ requires a conformation of the GlnB T-loop, which is stabilized by ADP, but is counteracted by joined ATP-Mg^2+^-2-OG binding (Llácer et al., 2010; Zeth et al., 2014; Lüddecke and Forchhammer, 2015). However, P_II_-PipX interaction is not affected by phosphorylation of P_II_ at S49 in the T-loop (Llácer et al., 2010). Formation of the P_II_-PipX complex prevents PipX to function as co-activator of the global nitrogen-transcription factor NtcA (Espinosa et al., 2006; Llácer et al., 2010). The interaction of P_II_ with NAGK is thought to be mediated in a two-step process (Ma et al., 2014). First, an encounter complex is formed, which leads in the second step to bending of the T-loop (Fokina et al., 2010b), enabling T-loop residues to interact with and activate NAGK (Llácer et al., 2007). This P_II_-NAGK interaction is highly sensitive to 2-OG, whose binding results in repulsion of the T-loop leading to the dissociation of the P_II_-NAGK complex. Complex formation with P_II_ strongly diminishes allosteric feedback-inhibition of NAGK by arginine (Maheswaran et al., 2004; Fokina et al., 2010a). 2-oxoglutarate is thought to be the key metabolite in signaling the carbon/nitrogen balance in cyanobacteria (Muro-Pastor et al., 2001). Lack of a nitrogen source or excess CO_2_ leads to an increase in the 2-OG pool (Muro-Pastor et al., 2001; Eisenhut et al., 2008; Hauf et al., 2013), which coincides with phosphorylation of GlnB (Forchhammer and Tandeau de Marsac, 1995a,b) The recent description of a highly conserved GlnB-BCCP interaction (Gerhardt et al., 2015) suggests that this interaction should also play a role in cyanobacteria. However, metabolic consequences of the GlnB-BCCP interaction have not yet been described and its physiological consequences remain unclear. A potential control of ACCase activity by P_II_ could link acetyl-CoA pools and synthesis of fatty acids to the nitrogen status of the cells. The levels of acetyl-CoA pools mirror its consumption through various acetyl-CoA dependent reactions and replenishment ultimately through CO_2_-fixation. Several studies have been performed in *Synechocystis* using metabolic engineering to increase acetyl-CoA pools (Liu et al., 2011; Tan et al., 2011). Yet, our understanding of the acetyl-CoA metabolism in cyanobacteria is limited. A few studies, that have addressed the question how acetyl-CoA pools respond during nitrogen deprivation came to controversial results: in some studies, the acetyl-CoA pools increased (Joseph et al., 2014; Anfelt et al., 2015), or were almost unchanged (Schlebusch and Forchhammer, 2010) whereas other reported modest (Osanai et al., 2014) or strong decrease (Hondo et al., 2015) upon nitrogen deprivation. Different growth conditions, extraction procedures, or data normalization could account for the divergence. So far, no study has been performed in which the acetyl-CoA pools during different growth conditions and C/N regimes were systematically compared in *Synechocystis*. This work was performed to verify the putative interaction of GlnB with BCCP in cyanobacteria and to reveal its physiological impact by studying acetyl-CoA metabolism and fatty acid accumulation in P_II_ mutant of *Synechocystis* sp. PCC 6803.

## Materials and methods

### Strains and plasmids

For all cloning procedures Q5 polymerase (NEB) was used. Constructs were assembled according to Gibson et al. (2009) from gBlocks® (IDT) or PCR products and the vector backbone. Sequence integrity was verified by DNA sequencing (GATC biotech). Bacterial strains and plasmids are listed in Table 3. Complementation of the P_II_ mutant was performed as described by Wolk et al. (1984) with plasmids pVZ322-P_II_-Ven and pVZ322-P_II_S49E-Ven.

### Protein expression

ACC of *E. coli* was extracted as described previously (Gerhardt et al., 2015). BirA was expressed in *E. coli* as described before (Gerhardt et al., 2015). P_II_ protein from *Synechocystis* and *Synechococcus* was purified as described previously (Heinrich et al., 2004). For expression of *Synechocystis* BCCP in *E. coli* BL21(DE3) was grown in 2YT medium at 37°C and expression was induced with IPTG at an OD_600_ of 0.8. Induced culture was cultivated at 25°C over night. Cells were harvested at 4000 × g for 10 min., cell pellets were combined with a cell suspension overexpressing BirA in biotinylation buffer (50 mM HEPES pH8, 10 mM KCl, 5% v/v glycerol, 5 mM MgCl_2_, 1 mM Biotin, 10 mM ATP, and 1 mM Benzamidine). Cells were homogenized with a Branson Sonifier S-250A and the lysate was incubated for 1 h at 37°C, followed by 4°C over night to biotinylate BCCP. BCCP was extracted from the cleared cell lysate (centrifugation for 30 min. at 25,000 × g at 4°C) through Ni-NTA affinity chromatography. Cleared lysate was loaded on a wash buffer (50 mM TrisHCl pH7.5, 100 mM KCl, 20% v/v glycerol, and 50 mM imidazol) equilibrated Histrap FF Crude column (GE healthcare). The column was washed with 10 column volumes wash buffer and bound protein was eluted with elution buffer (50 mM TrisHCl pH7.5, 100 mM KCl, 20% v/v glycerol and 500 mM imidazol). The eluted protein was dialyzed against a storage buffer (50 mM HEPES pH 7.8 100 mM KCl, 50% v/v glycerol) over night at 4°C. Biotinylation of BCCP was verified using immunoblotting and subsequent detection of biotinylated proteins using streptavidin-HRP conjugate with chemiluminscence.

### ACC activity

ACC activity was measured by coupling ACC catalyzed ATP hydrolysis to the activities of pyruvate kinase (PK) and lactate dehydrogenase (LDH) as described (Beez et al., 2009; Broussard et al., 2013). The reaction buffer consisted of 50 mM imidazole, 50 mM KCl, 20 mM MgCl_2_, 0.2 mM NADH, 1 mM phosphoenolpyruvate, 10 mM ATP, 0.5 mM DTT, 4.4 units of LDH, 6 units PK, and 10 mM NaHCO_3_. The pH of the final reaction mixture was 7.5. Following concentrations of ACC subunits were used for the enzyme assay: 10 nM carboxyl transferase (tetramer), 20 nM biotin carboxylase (dimer) and 200 nM biotin carboxyl carrier protein (monomer). Different concentrations of P_II_ and 2-OG were used as indicated in the text. The reactions were pre-incubated for 15 min. and started by the addition of acetyl-CoA 400 μM. The oxidation of NADH to NAD^+^ was recorded at 25°C over 20 min. in a SPECORD 200 photometer (Analytik Jena) at 340 nm. From the slope of decreasing absorption, reaction velocity was calculated with an extinction coefficient for NADH of 6220 M^−1^. For the determination of catalytic constants, the data were fitted to Michaelis-Menten equation using GraphPad prism software

### Protein co-precipitation

Prior to protein co-precipitation experiments BCCP conformation was checked by size exclusion chromatography (20 mM potassium phosphate buffer pH 7.8 100 mM NaCl) ensuring properly folded BCCP was used for experiments. 30 μl Ni-NTA agarose coated magnetic beads (Quiagen) preequilibrated in binding buffer (50 mM TrisHCl pH 8.0, 100 mM NaCl, 0.1% w/v N,N-Dimethyldodecylamine N-oxide (LDAO), 10% v/v glycerol, and 20 mM imidazole) were used. Binding was performed in 700 μl binding buffer with magnetic beads, 30 μg BCCP, and 35 μg P_II_ for 20 min. at room temperature. Unbound protein was washed off, three times with 300 μl binding buffer and bound proteins were eluted in 20 μl elution buffer (50 mM TrisHCl pH 8.0, 100 mM NaCl, 0.1% w/v N,N-Dimethyldodecylamine N-oxide (LDAO), 10% v/v glycerol, and 500 mM imidazole). Various metabolites were added to the binding buffer with final concentrations as indicated. Eluted fractions were analyzed by Tricine-SDS PAGE (Schägger, 2006) and stained with InstantBlue (Expedeon). Stained gels were scanned and band intensities were analyzed densitometrically. Scanned images were gray scaled and inverted with Adobe PhotoshopCS6, mean pixel intensities were determined for the P_II_ protein band and used as a proxy for protein abundance for subsequent analysis.

### Cyanobacterial cultivation

*Synechocystis* sp. PCC 6803 was grown in BG11 medium (Rippka et al., 1979) at 27°C, supplemented with 5 mM NaHCO_3_ on a rotary shaker at light intensities of 50–80 μmol photons s^−1^m^−2^. For imposing nitrogen-starvation conditions, cells were first grown in BG11 medium to an optical density (750 nm) of 0.4–0.6, harvested by centrifugation at 4000 × g for 10 min., then washed with BG11-N (BG11 lacking NaNO_3_), pelleted again at 4000 × g for 10 min., and finally re-suspended in BG11–N (supplemented with 5 mM NaHCO_3_) to an OD_750_ of 0.4. For growth with ammonium, cells were grown to OD_750_ of 0.6–0.8 and diluted in BG11-N medium to OD_750_ 0.1. The medium was buffered with TES pH8, supplemented with NaHCO_3_ and NH_4_Cl to a final concentration of 5 mM.

### Estimation of intracellular acetyl-CoA

To estimate the intracellular acetyl-CoA levels 20 ml of growing culture was pelleted at 4000 × g for 10 min. and frozen at −80°C until measured. Cell pellets were suspended in 200 μl 1 M cold perchloric acid. Suspended cells were lysed using a FastPrepR-24 (MP Biomedicals) for 30 s and 6.5 m/s five times with glass beads (0.1–0.11 mm diameter). Cell debris and glass beads were pelleted at 13,000 × g at 4°C for 10 min. The supernatant was neutralized with 3 M KHCO_3_ and excess KHCO_3_ was removed through centrifugation at 13,000 × g for 2 min. at 4°C. The clear supernatant was used for acetyl-CoA measurements using the Acetyl-CoenzymeA kit (Sigma-Aldrich) according to the manufacturer's instruction. Fluorescence intensities were measured using a SpectraMax M2 microplate reader with λ_ex_ = 535 nm and λ_em_ = 587 nm.

### Fatty acid quantification

Fatty acids were quantified as described previously (Wawrik and Harriman, 2010). Cell pellets of 2 ml culture were thawed in in 200 μl saponification reagent (25% methanol in 1N NaOH) and lysed with glass beads (0.1–0.11 mm diameter) in a FastPrepR-24 (MP Biomedicals) for 30 s and 6.5 m/s five times. Cell lysates were saponified for 30 min. at 95°C and vortexed every 5 min. Cell extracts were neutralized with 200 μl neutralization reagent (1N HCl, 100 mM Tris pH 8.0) and copper reagent (9 vol. aq. 1 M triethanolamine, 1 vol. N-acetic acid, 10 vol. 6.45% (w/v) Cu (NO_3_)_2_·3H_2_O). Samples were vortexed for 2 min. and 250 μl chloroform was added and vortexed for additional 2 min. Phase separation was achieved by centrifugation and 50 μl of the organic phase was transferred in two separate new tubes. In one tube 50 μl 2-butanol were added and used as blank. The second tube was mixed with 1% (w/v) sodium diethyldithiocarbamate in 2-butanol leading to color development in the sample. Absorption was measured at 440 nm in a SpectraMaxM2 microplate reader and the absorption of the blank was subtracted from the sample manually. Lipid concentration was estimated based on a standard curve with palmitic acid.

### Fatty acid composition

Two hundred milliliters exponentially growing culture were harvested at 4000 × g at 25°C, the cell pellet was washed once with water, pelleted at 20,000 × g for 3 min., frozen in liquid nitrogen and stored at −80°C until used. Cell pellets were lyophilized for 16 h. Pentadeconoic acid was added to 20 mg CDW which was used for saponification with 1 ml 3,75 M NaOH in 50% methanol (v/v) for 35 min. at 100°C. Free fatty acids were methylated by addition of 2 ml methylation reagent (3.25 M HCl in 45% methanol (v/v) for 12 min. at 80°C. Fatty acid methyl esters (FAME) were extracted with 2 ml n-hexane through vortexing and 10 min. incubation on a revolving laboratory mixer. The organic phase was transferred in a new vial to which 3 ml 0.3 M NaOH were added and incubated for 10 min. on a revolving laboratory mixer. The organic phase was transferred in a GC vial and evaporated under nitrogen gas flow at 60°C. FAME were dissolved in 50 μl dichlormethane and analyzed by gas chromatography.

### GC analysis of fatty acid methyl esters

GC analysis was performed with a Shimadzu GC9A equipped with a FID detector and a DBWAX-30 W (30 m × 0.319 mm) column with nitrogen as carrier gas. 5 μl of sample was injected, the injector and detector temperature was set at 250°C. The Oven temperature increased from 160° to 200°C at a rate of 4°C per minute, and from 200 to 240°C at 8°C per minute and remained constant for 10 min. at 240°C. Fatty acid methyl esters were identified based on retention times determined with commercially available fatty acid methyl esters. FAME were quantified using response factors with pentadecanoic acid as internal standard.

### Extraction of lipids from cellular biomass

Two hundred milliliters exponentially growing culture was pelleted at 4000 × g for 10 min. at 25°C. The pellet was washed with deionized water, cells were pelleted at 20,000 × g for 3 min. and the pellet was frozen at −20°C until further use. Bacterial pellets were dried in a centrifugal evaporator for 16 h at 25°C. Dried cell matter (15–40 mg CDW) was used for lipid extraction as described before (Bligh and Dyer, 1959). Dried material was transferred in a glass vial with a PTFE lined screw cap lid, suspended in 3 ml Methanol:Chloroform (2:1), vortexed vigorously and incubated for 1 h on a revolving laboratory mixer. After incubation 1 ml chloroform and 1.8 ml deionized water were added, vortexed and phase separation was induced through centrifugation for 10 min. at 4000 × g. The organic phase was transferred in a fresh glass vial and the aqueous phase was extracted twice with 1 ml chloroform followed by 4 ml Isooctane:Ehtylacetate (3:1). All organic phases were combined and solvents were evaporated under nitrogen gas stream. Lipids were suspended in either 200 μl Chloroform: Methanol (1:1) or Hexane:Ether:Acetic acid (80:20:1).

### Lipid droplet visualization in synechocystis

To 100 μl *Synechocystis* cell suspension 1 μl Bodipy® 493/503 (10 mg/ml in DMSO) was added and incubated for 5 min. Cells were pelleted at 10,000 × g for 2 min. and cell pellets were suspended in PBS buffer pH 7.5. Two microliter were dropped on a poly lysine coated glass slide and examined using a Leica DM5500B microscope. Image acquisition was performed with a Leica DFC360FX black and white camera, fluorescence images were recolored using Leica application suite. Green fluorescence was detected using an excitation filter BP470/40 and an emission filter BP525/50. Fluorescence images were acquired with 100 ms exposure time. Bright field images were acquired with 6 ms exposure time. Intensity levels of images were adjusted using PhotoshopCS6.

### TLC of lipids

Lipid extracts were spotted on silica gel 60 (Merck Millipore) TLC plates. Phospholipids were resolved using Chloroform:Methanol:NH_4_OH (70:30:5) as mobile phase (Merritt et al., 1991). Glycolipids were visualized spraying the plates with 2.4% (w/v) α-naphtol in 10% sulfuric acid 80% (v/v) ethanol and baking the plate at 120°C until purple spots were visible (Wang and Benning, 2011). Neutral lipids were resolved using a Hexane:Ether (90:10) mobile phase and stained with iodine vapor (Ruiz-Lopez et al., 2003). Individual spots were scraped of and lipids were extracted with Hexane:Ether:Acetic acid (80:20:1).

### GC/MS analysis

Solvent extracted lipids from silica gel were subjected to saponification and FAME were synthesized as described above. FAMEs were detected using a Shimadzu GC17A with a QP-5000MS (GC-MS) using an optima 5MS (15 m × 0.25 mm) column with Helium as carrier gas. 5 μl of sample was injected, the injector temperature was set at 320°C. The column was heated to 90°C and the temperature was hold for 5 min., heated up at a rate of 20°C/min. to 200°C, heated at a rate of 4°C/min. to 300°C and hold for 2 min. at 300°C. The MS detector voltage was set at 1.65 keV.

## Results

### Cyanobacterial GlnB affects the activity of *E. coli* ACC

**Figure 1:**
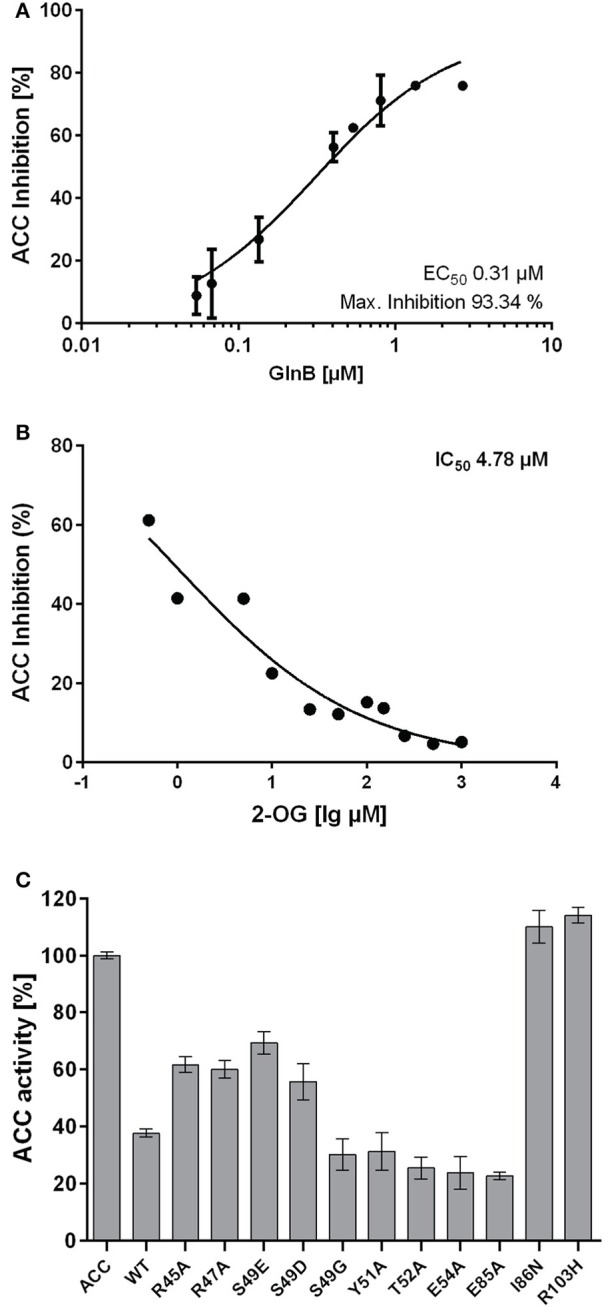
**(A)** Inhibition of ACC activity in response to various GlnB concentrations. The EC_50_ value was calculated to be 0.31 μM (SE: 0.07 μM). **(B)** Inhibition of ACC with increasing 2-OG concentrations. The IC_50_ was calculated to be 4.8 μM (SE: 0.2 μM). **(C)** Activity of ACC with various GlnB point mutated proteins present in the reaction mixture.

The *E. coli* acetyl-coenzyme A carboxylase (ACC) was used in a previous study as a model system to investigate the effect of GlnB/GlnZ from *Azospirillum brasilense* and GlnB/GlnK from *E. coli* on enzyme activity (Gerhardt et al., 2015). Here, we first investigated the effect of several characterized *Synechococcus elongatus* PCC7942 P_II_ protein variants (*Sc*P_II_) on ACC activity. Initial assays were carried out at a fixed concentration of 10 mM ATP. *Synechococcus* GlnB (*Sc*GlnB) was able to efficiently inhibit the *E. coli* ACC activity and increasing concentrations of GlnB correlated with increased inhibition of ACC (Figure 1A). The maximum inhibition was calculated to be 93% (SE: 6.8%) with an EC_50_ for *Sc*GlnB of 0.31 μM (SE: 0.066 μM). As interaction of BCCP and *Azospirillum* GlnB was shown to be affected by 2-OG, ACC activity was measured in presence of 1 μM GlnB and various 2-OG concentrations (Figure 1B). Increasing concentrations of 2-OG were able to efficiently relief ACC from GlnB-dependent inhibition and the IC_50_ value for 2-oxoglutarate was calculated to be 4.8 μM (SE: 0.2 μM), which is almost exactly the K_d_ of the first 2-OG binding site (5.1 μM) of GlnB (Fokina et al., 2010b). To reveal, which positions in *Sc*P_II_ are important for ACCase regulation, various variants of *Sc*P_II_ were tested in their ability to inhibit ACC activity (Figure 1C). Point mutations in the T-loop of R45 and R47 to alanine and the phosphomimetic S49D/S49E variants were not as efficient in inhibiting ACC activity as wild type P_II_. In contrast, mutations of S49G, Y51A, and E54A in the T-loop and E85A were not affected in inhibiting ACC activity. Two P_II_ variants (I86N and R103H) were, however, completely unable to inhibit ACC activity. Addition of 1 mM 2-OG to the reaction relieved ACC inhibition in all P_II_ variants. This confirms the previous assumption, that GlnB regulation of ACCase activity is highly conserved in bacteria.

### SyGlnB-BCCP interaction depends on the concentration of ATP and 2-OG

**Figure 2:**
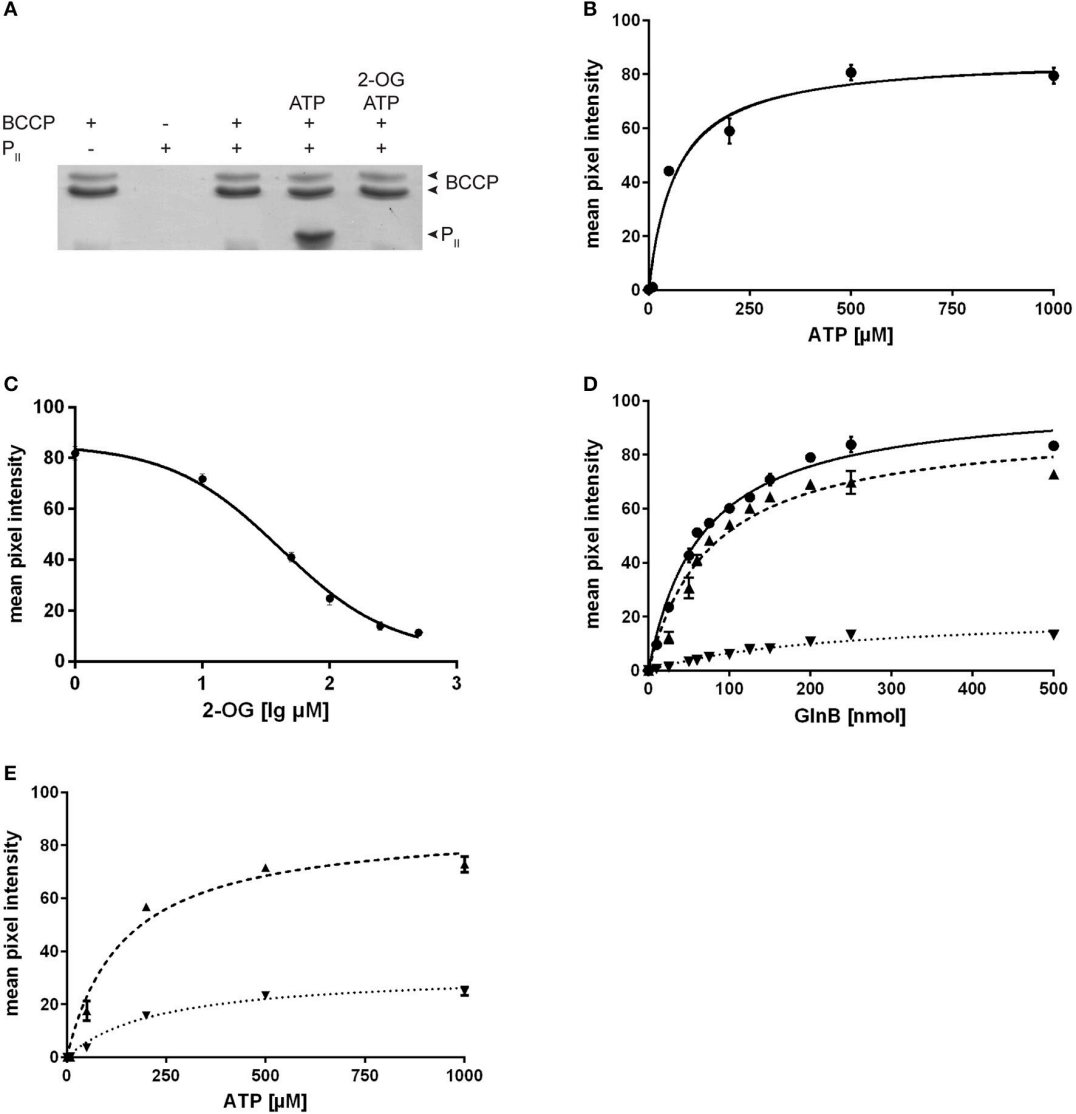
**(A)** Pull down experiments of Synechocystis BCCP and GlnB using BCCP as bait. Experiments were performed using 0.5 mM Mg^2+^, 0.5 mM ATP and 1 mM 2-OG where indicated. **(B)** ATP titration of the BCCP-GlnB interaction while the amount of GlnB used was held constant and a calculated EC_50_ value of 68.0 μM (SE 13.2 μM) for ATP. The maximum binding was calculated to be 86.6 mpi (SE 3.8 mpi). **(C)** Influence of 2-OG on the BCCP-GlnB interaction. The IC_50_ of 2-OG is 41.3 μM (SE 1.7 μM). **(D)** Increasing amounts of GlnB and its S49 variants were used with BCCP as bait with 0.5 mM Mg^2+^ and 0.5 mM ATP. The EC_50_ is 65.9 nmol (SE 4.2 nmol) for wild type (circles), 77.0 nmol (SE 8.6 nmol) for S49C (triangles) and 224 nmol (SE 32.1 nmol) for S49D (inverted triangles). Maximum binding was calculated to be 100.7 mpi (SE 2.2 mpi) for the wild type protein, 91.4 mpi (SE 3.7 mpi) for the S49C variant and 21.0 mpi (SE 1.6 mpi) for the S49D variant. **(E)** ATP titration of the BCCP-GlnB S49C and S49D variant (as in **B**). The calculated ATP EC_50_ value for S49C variant is 143 μM (SE 24 μM) and 231 μM (SE 36 μM) for the S49D variant. Maximum binding was calculated to be 88.2 mpi (SE 3.8 mpi) for the S49C variant and 32.2 mpi (SE 1.7 mpi) for the S49D variant.

Since the aim of this study was to characterize the physiological effect of P_II_ on the acetyl-CoA metabolism, but the P_II_ mutant of *Synechococcus* accumulates second site mutations in *pipX* (Espinosa et al., 2009) we decided to study this effect in the P_II_ mutant of *Synechocystis*, in which *pipX* and *ntcA* are not affected. Even though *Sc*GlnB shares 95% sequence identity with GlnB of *Synechocystis* (*Sy*GlnB) we wanted to verify the interaction of *Sy*GlnB and *Synechocystis* BCCP proteins *in vitro*. To this end, recombinant proteins were expressed and purified from *E. coli*. His-tagged BCCP was used as bait protein using Ni-NTA coated magnetic beads. BCCP GlnB interaction was strictly dependent on the presence of Mg^2+^ ions and ATP. Like in *A. brasilense* and *E. coli*, 2-OG negatively affected the ATP-dependent P_II_ binding to BCCP (Figure 2A). No P_II_ protein could be recovered in the presence of ADP. An ATP titration experiment was performed and the amount of co-precipitated protein was plotted against the ATP concentration (Figure 2B). The apparent EC_50_ for ATP was determined to be 68 μM (SE 13.2 μM) through non-linear fitting and is in good agreement with the K_*d*_ of the third ATP binding site of cyanobacterial GlnB (47.4 μM), which exhibits three anticooperative sites (Fokina et al., 2010b). The same type of analysis was performed for 2-OG, titrated in the presence of a fixed concentration of 0.5 mM ATP (Figure 2C). The apparent IC_50_ value was calculated, assuming dose response dependent inhibition using a standard slope. The resulting IC_50_ for 2-oxoglutarate was determined to be 41.3 μM (SE 1.7 μM). This value is lower than the K_*d*_ of the third GlnB 2-OG binding site (106.7 μM) but well above the Kd of the second site (11.1 μM) (Fokina et al., 2011), which suggests that occupation of the third 2-OG binding determines dissociation of the *Sy*GlnB-*Sy*BCCP complex. GlnB is known to be phosphorylated *in vivo* at position Ser49 under nitrogen-poor conditions or high CO_2_-supply to nitrate-grown cells. In the case of P_II_-NAGK interaction, Ser49 phosphorylation prevents complex formation (Heinrich et al., 2004) and the phosphomimetic variant S49D was unable to interact with NAGK (Llácer et al., 2007). As shown above, the phosphomimetic variants of *Sc*GlnB (S49D and S49E) had reduced efficiency in inhibiting *E. coli* ACCase. To find out, how phosphomimetic variants *Sy*GlnB are affected in binding the cognate *Sy*BCCP protein, the affinity of *Sy*GlnB variants S49D, S49E, S49C, and the wild type protein were tested toward *Sy*BCCP through pull down experiments (Figure 2D). Instead of using the S49G variant we decided to use the S49C variant as mutation of S49 to glycine could have a negative impact on complex stability (Lüddecke and Forchhammer, 2013). The S49E variant was completely unable to bind BCCP. The other negatively charged variant S49D, weakly interacted with BCCP, showing only about 20% maximal binding as compared to wild-type GlnB. Likewise, the EC_50_ for GlnB increased 4-fold compared to wild type GlnB. By contrast, substitution of Ser49 to Cys had only a minor effect on GlnB-BCCP interaction (about 90% maximal binding), indicating that the negative charge at position 49 that impairs BCCP-GlnB interaction. As ATP binding influences the T-loop conformation, a titration of ATP with the two variants S49C and S49D was performed (Figure 2E). The S49C mutation increases the calculated EC_50_ value for ATP from 68 to 143 μM (SE 24 μM) and to 231 μM (SE 36 μM) for the S49D variant. Maximum binding of GlnB was calculated to be 88.2 mean pixel intensity (SE 3.8 mpi) for the S49C variant which was almost identical to the wild type protein (86.6 mpi; SE 3.8 mpi), but was much lower for the S49D variant with 32.2 mpi (SE 1.7 mpi) at saturating ATP concentrations. On the one hand, the doubling of the EC_50_ for ATP implies that substitution of serine 49 to cysteine (which is bulkier) requires increased ATP concentrations to fit the T-loop into a conformation that binds to BCCP. At excess ATP concentrations, the mutation had no influence on the total amount of GlnB that can be co-precipitated with BCCP, in agreement with the GlnB titration experiment above. On the other hand, when the T-loop carries the S49D mutation, more than three times higher ATP concentrations were required to enforce the appropriate conformation for complex formation with BCCP. Moreover, the stability of the complex was reduced to one third, as compared to the complex with wild-type GlnB. Taken together, introduction of a negative charge at position 49 in the T-loop of P_II_ destabilizes the BCCP-GlnB complex. This, together with the fact, that the S49E *Sy*GlnB variant was completely unable to interact with BCCP, strongly indicates that phosphorylated P_II_ will not be able to interact with BCCP.

### Deletion of *GlnB* alters acetyl-CoA metabolism

**Figure 3:**
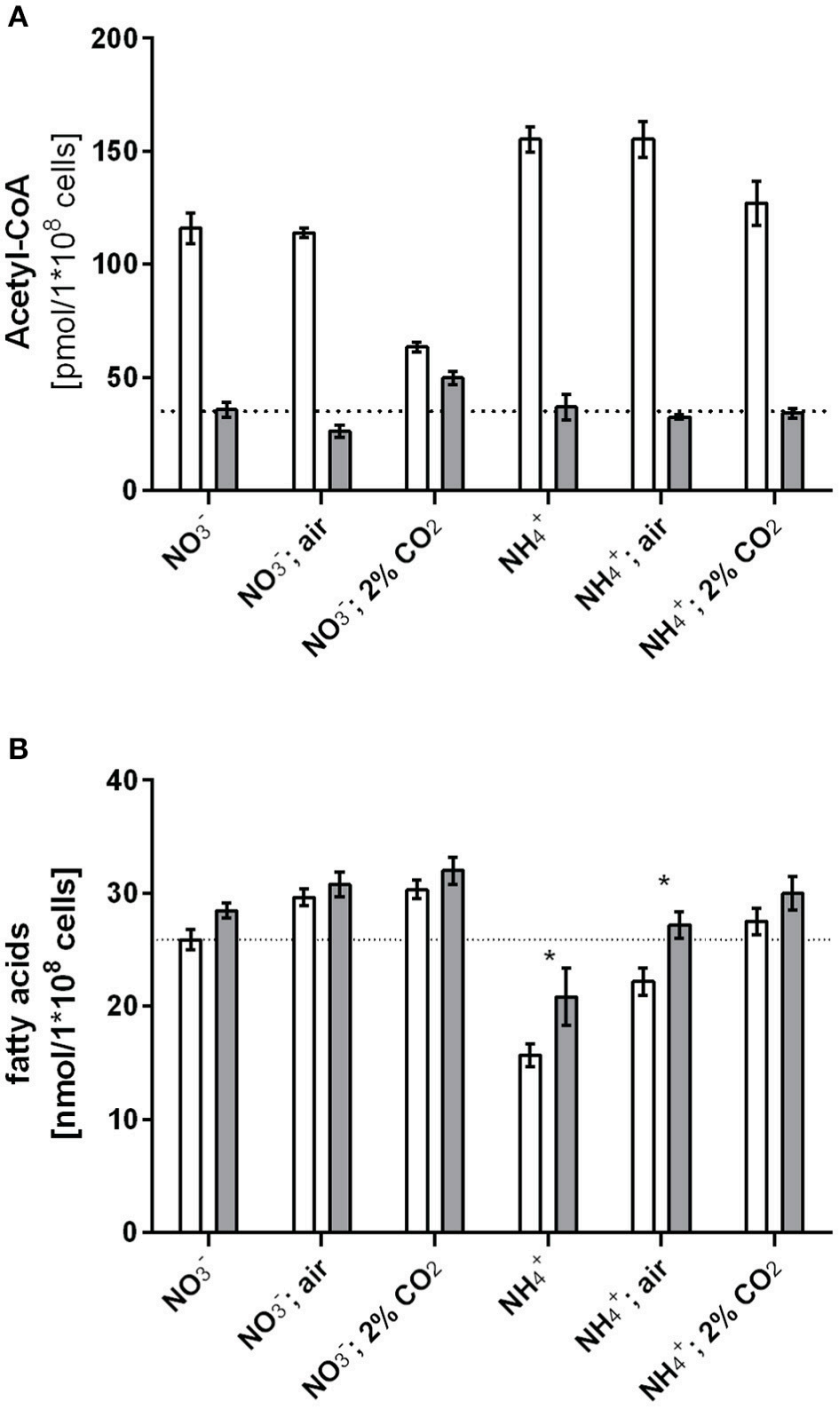
**Acetyl-CoA levels (A) and fatty acid levels (B) under different carbon nitrogen regimes during exponential growth in the wild type and the P_**II**_ mutant**. Values represent the mean of three biological replicates. Differences in acetyl-CoA levels using the tested growth conditions are statistically significant (*p* < 0.05; unpaired *t*-test). Statistically significant values of fatty acid levels are marked with a star (*p* < 0.05; unpaired *t*-test).

The *in vitro* experiments showed that GlnB directly binds BCCP and affects ACC activity, and furthermore, interaction is sensitive to Ser49 modification. From these findings, we hypothesized that phosphorylation of P_II_ should have an impact on either fatty acid or acetyl-CoA metabolism during varying carbon-nitrogen regimes, which correspond to different degrees of P_II_ phosphorylation in *Synechocystis* (Forchhammer and Tandeau de Marsac, 1995a). This regulation should be abolished in a *Synechocystis* P_II_ mutant. To examine this prediction, wild type and P_II_ mutant strains were grown with different nitrogen and carbon supply (nitrate or ammonia as nitrogen source, gently shaking without aeration, corresponding to the lowest CO_2_ supply; or vigorous bubbling with either ambient air (0.04%) or 2% CO_2_). The expected phosphorylation status of P_II_ was verified (Supplementary Figure 1) and cellular acetyl-CoA levels as well as total fatty acid concentrations were determined in exponentially growing cultures under these conditions. Regardless of the carbon or nitrogen regime, the acetyl-CoA level in the P_II_ mutant was always much lower than in the wild type (Figure 3A). Remarkably, the acetyl-CoA levels in the wild type differed with changing carbon and nitrogen regimes. In particular in nitrate grown cells, the acetyl-CoA levels decreased significantly in presence of 2% CO_2_ supply. Under these conditions, P_II_ displays the highest degree of phosphorylation, and acetyl-CoA levels in wild-type and mutant cells are similar. However, under any condition that leads to a low degree of P_II_ phosphorylation (either nitrate grown with limiting CO_2_-supply or ammonia grown cells), the acetyl-CoA levels were strongly increased, whereas it stayed low in the P_II_ deficient mutant. Total fatty acid levels did not differ as much as the acetyl-CoA levels, but slightly higher fatty acid levels in the P_II_ mutant were always visible. The differences were particularly significant in ammonia grown cells with low carbon supply, where P_II_ is always present in the non-phosphorylated state in the wild type (Figure 3B). The carbon regime had a marked impact on the fatty acid content, in both strains. Increased CO_2_ supply favored a higher intracellular lipid content. This effect is probably due to improved CO_2_-fixation, that will ultimately result in increased CO_2_-fixation products than can flow into various anabolic pathways.

**Figure 4:**
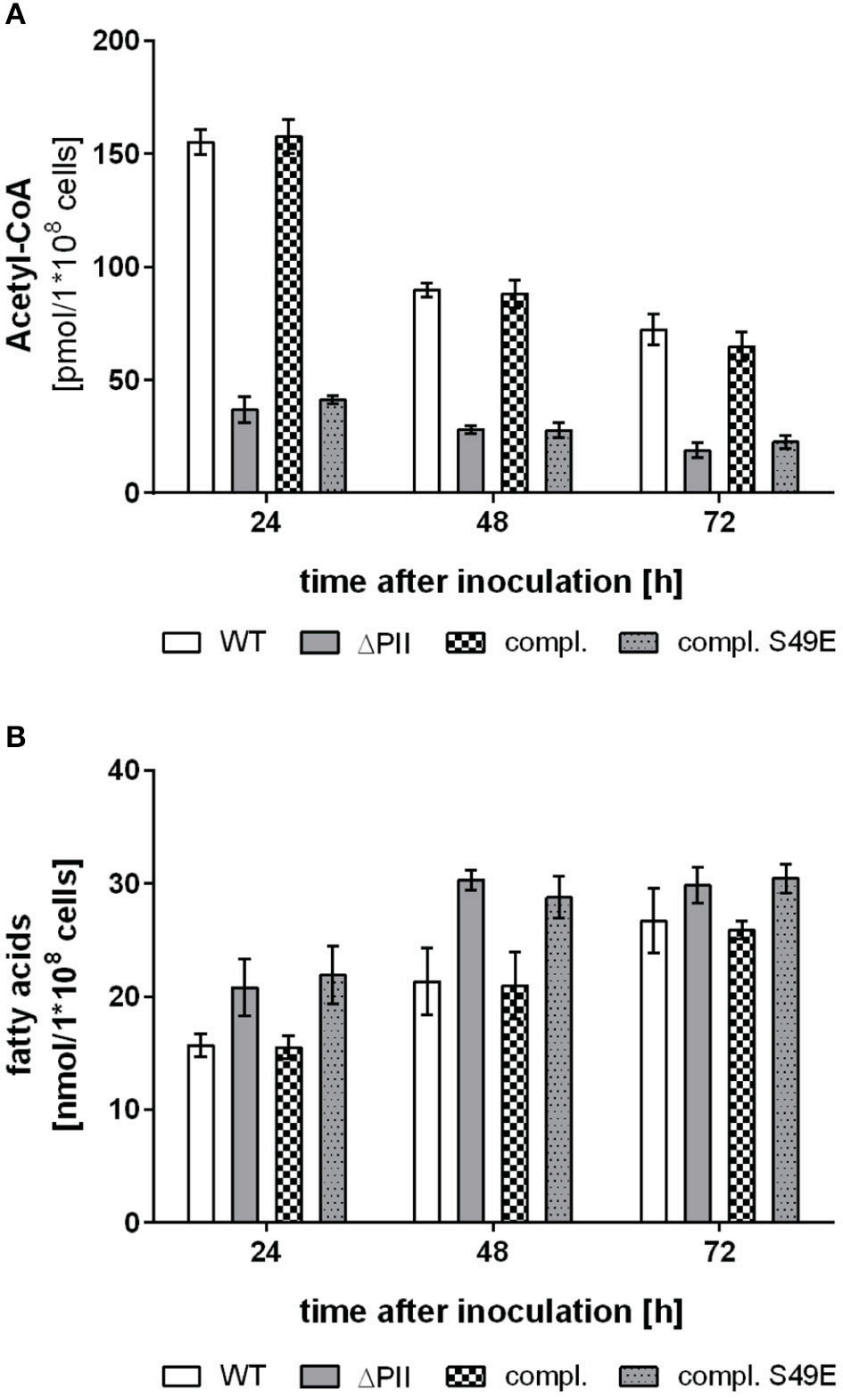
**Accumulation of acetyl-CoA (A) and fatty acids (B) during growth in standard BG11 with ammonia as nitrogen source in wild type (white bars), the P_**II**_ mutant (gray bars), the P_**II**_ complemented strain (checked bars) and the P_**II**_-S49E complemented strain (gray dotted bars)**. Values represent the mean of three biological replicates. Differences in acetyl-CoA levels are statistically significant throughout growth between wild type and the P_II_ mutant (*p* < 0.05; unpaired *t*-test). Differences in fatty acid levels between wild type and the P_II_ mutant are statistically significant within the first 48 h of growth (*p* < 0.05; unpaired *t*-test).

Nitrogen starvation represents the situation of maximal P_II_ phosphorylation (Forchhammer and Tandeau de Marsac, 1995a). If the assumption is correct, that P_II_ phosphorylation abrogates its inhibitory effect on ACCase, then the differences in acetyl-CoA levels between wild-type and P_II_ mutant should disappear under those conditions. Therefore, we analyzed acetyl-CoA and total fatty acid levels of cells subjected to 8 h nitrogen-starvation and compared it to conditions during exponential growth with nitrate as nitrogen source. In agreement with our expectation, in 8 h nitrogen-starved cells, the acetyl-CoA levels dropped in the wild-type to the low levels observed in the P_II_ mutant (Table 1). As acetyl-CoA levels in *E. coli* decrease during late exponential (Chohnan and Takamura, 1991) and stationary phase, the growth phase dependence of acetyl-CoA levels was measured in *Synechocystis* strains. In the wild-type, acetyl-CoA levels were high during exponential growth and decreased with increasing optical densities. As already shown above, strongly reduced levels of acetyl-CoA in the P_II_ mutant were visible over all time points (Figure 4A). Complementation of the P_II_ mutant with the wild-type *glnB* gene was able to complement the low acetyl-CoA level phenotype, but introduction of the gene encoding the P_II_ S49E variant, which was not able to interact with BCCP retained the mutant phenotype. Total fatty acid levels generally increased during growth and the difference between wild type and P_II_ mutant got smaller at the later stages of growth but was significantly different in the first 48 h of growth (Figure 4B). The complemented strain had similar fatty acid levels as the wild type, but the S49E variant was not able to complement the P_II_ mutant phenotype. The difference between wild-type and P_II_ mutant in fatty acid levels during ammonia-supplemented growth was verified using GC analysis. GC results matched the values obtained with the colorimetric assay but additionally provided qualitative information, how fatty acid composition might be altered. As shown in Table 2, mutation of GlnB shifted the molar composition of fatty acids, which increased the amount of palmitic acid by about 15% at the same time decreasing the amount of linoleic acid to the same extent. The fatty acid profile of the P_II_ complemented strain was very similar to that of the wild type, whereas the S49E strain had a fatty acid composition reminiscent of the P_II_ mutant exemplifying that the S49E P_II_ variant is a loss-of function mutant with respect to regulation of fatty acid metabolism. Triple unsaturated fatty acids were increased in both complemented strains.

**Table 1:** **Acetyl-CoA and total fatty acid levels of wild type and the P_**II**_ mutant during exponential growth and 8 h after nitrogen starvation**.

	**Wild type**	**ΔP_II_**	**Wild type**	**ΔP_II_**
	Acetyl-CoA [pmol/1^*^10^8^ **cells]**		Fatty acids [nmol/1^*^10^8^ **cells]**	
0 h	127.45 ± 9.3	43.95 ± 3.4	22.37 ± 1.2	25.43 ± 1.7
8 h	47.74 ± 2.0	48.98 ± 1.5	18.92 ± 2.4	21.15 ± 1.0

**Table 2:** **Molar composition of fatty acids in %**.

	**C16**	**C16:1**	**C18**	**C18:1**	**C18:2**	**C18:3**
Wild type	26.86 ± 0.05	10.36 ± 0.19	1.94 ± 0.8	1.01 ± 0.28	56.68 ± 0.52	3.15 ± 1.0
ΔP_II_	42.46 ± 5.92	10.92 ± 0.78	1.26 ± 0.52	2.85 ± 0.37	41.37 ± 4.67	1.13 ± 0.4
compl.	26.82 ± 3.81	10.06 ± 1.12	3.24 ± 0.72	1.21 ± 0.17	46.52 ± 11.92	12.14± 7.34
compl. S49E	38.04 ± 7.27	8.96 ± 1.31	1.54 ± 0.25	1.74 ±0.29	37.73 ± 7.39	11.99 ± 9.32

*Values represent mean values and SE of at least three biological replicates*.

**Table 3:** **Bacterial strains and plasmids used in the study**.

**Strain/plasmid**	**Genotype/description**	**Source/reference**
**STRAINS**		
*E. coli* Top10	General cloning strain	Invitrogen
*E. coli* BL21 (DE3)	Strain for protein expression	Invitrogen
*E. coli* J53 (RP4)	Helper strain for tri-parental mating	Wolk et al., 1984
*Synechocystis* sp. PCC6803	Wild type strain	Stanier et al., 1971
ΔP_II_	*glnB−* strain of *Synechocystis* sp. PCC6803	Hisbergues et al., 1999
Complementation	ΔP_II_ strain complemented with P_II_-Venus	This study
Complementation S49E	ΔP_II_ strain complemented with P_II_S49E-Venus	This study
**PLASMIDS**		
pET15b	Expression vector for His-tagged proteins	Novagen
pET15*accB*	Expression of *Synechocystis* His-BCCP	This study
pCY216	Expression of *E. coli* BirA	Chapman-Smith et al., 1994
pET16b*accAD*	Expression of *E. coli* His-AccA and AccD	Soriano et al., 2006
pET16b*accC*	Expression of *E. coli* His-AccC	Soriano et al., 2006
pTRPETBCCPn	Expression of *E. coli* His-BCCP	Rodrigues et al., 2014
pASK-IBA3	Expression vector for Strep-taged proteins	IBA life sciences
pASK-IBA3*glnB*	Expression of C-terminally tagged GlnB from *Synechococcus elongatus*. PCC7942	Heinrich et al., 2004
pASK-IBA3*glnBS49D*	*Synechococcus* GlnB variant S49D	Espinosa et al., 2006
pASK-IBA3*glnBS49E*	*Synechococcus* GlnB variant S49E	Heinrich et al., 2004
pASK-IBA3*glnBR45A*	*Synechococcus* GlnB variant R45A	This study
pASK-IBA3*glnBR47A*	*Synechococcus* GlnB variant R47A	This study
pASK-IBA3*glnBS49G*	*Synechococcus* GlnB variant S49G	This study
pASK-IBA3*glnBY51A*	*Synechococcus* GlnB variant Y51A	This study
pASK-IBA3*glnBT52A*	*Synechococcus* GlnB variant T52A	This study
pASK-IBA3*glnBE54A*	*Synechococcus* GlnB variant E54A	This study
pASK-IBA3*glnBE85A*	*Synechococcus* GlnB variant E85A	This study
pASK-IBA3*glnBI86N*	*Synechococcus* GlnB variant I86N	Fokina et al., 2010b
pASK-IBA3*glnBR103H*	*Synechococcus* GlnB variant R103H	This study
pASK-IBA3*glnBSc*	Expression of C-terminally tagged GlnB from *Synechocystis* sp. PCC6803	This study
pASK-IBA3*glnBS49CSc*	*Synechocystis* GlnB variant S49C	This study
pASK-IBA3*glnBS49DSc*	*Synechocystis* GlnB variant S49D	This study
pASK-IBA3*glnBS49ESc*	*Synechocystis* GlnB variant S49E	This study
pVZ322	Broad host range expression vector	Grigorieva and Shestakov, 1982
pVZ322-P_II_-Ven	Expression of wild type GlnB with the fluorophore Venus at the C-terminus	This study
pVZ322-P_II_S49E-Ven	Expression of GlnB S49E variant with the fluorophore Venus at the C-terminus	This study

### Altered acetyl-CoA metabolism promotes intracellular lipid accumulation

**Figure 5:**
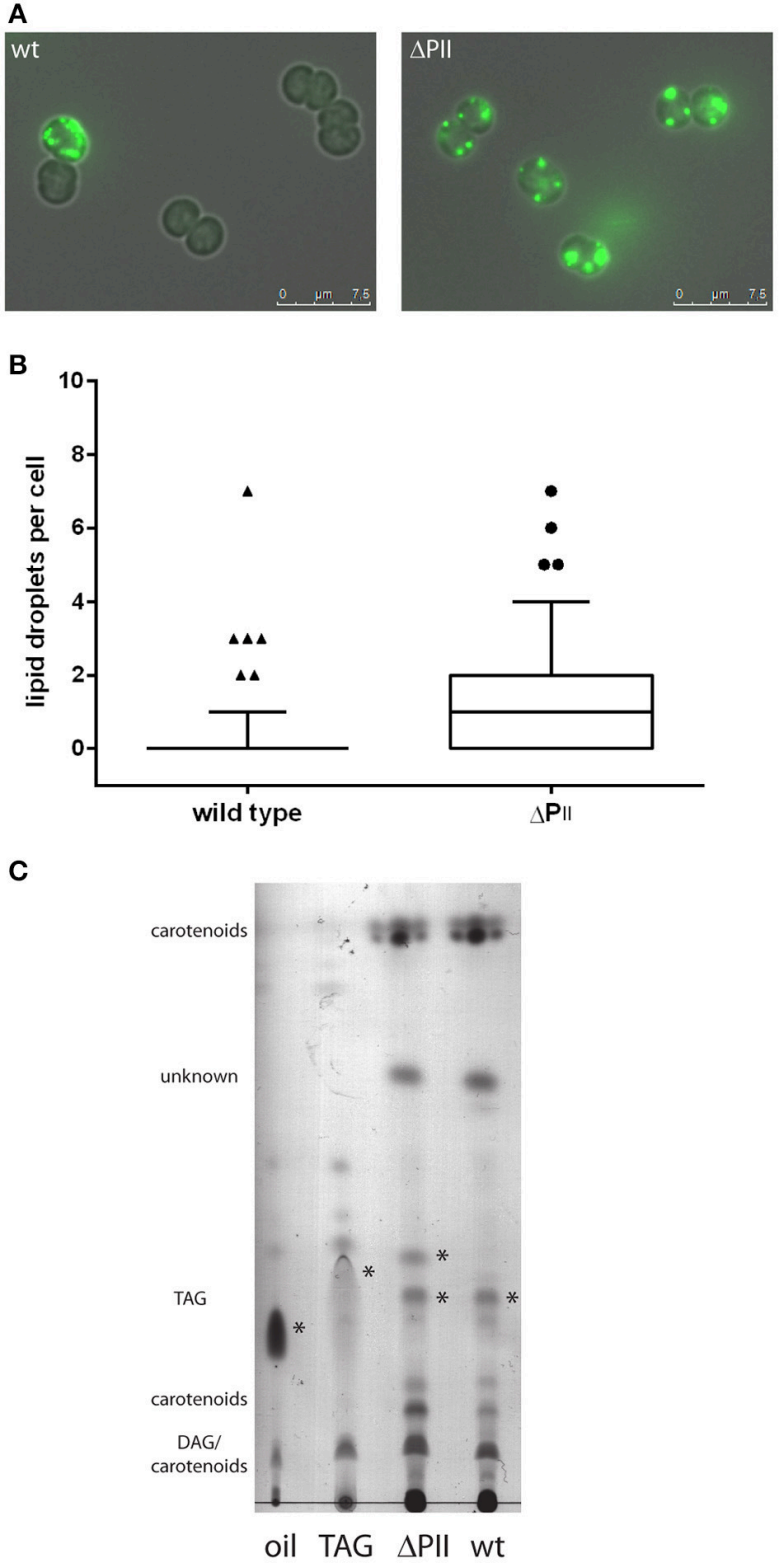
**(A)** Microscopic image of wild type and P_II_ mutant stained with Bodipy 493/503 during exponential growth. Images are the superimposition of the fluorescence and bright field image. **(B)** Occurrence of lipid droplets per cell in the wild type (mean: 0.39 ± 0.12) and P_II_ mutant (mean: 1.65 ± 0.23) at an OD_750_ of 0.2. The box plot displays the 10–90 percentile. The result is statistically significant with a *p*-value smaller than 0.0001 determined by an unpaired two tailed *t*-test. **(C)** Thin layer chromatography of hydrophobic lipids. Possible triacylglycerols are marked with asterisk. *DAG: diacylglycerol TAG: triacylglycerol*.

Intracellular lipids can be visualized microscopically using the hydrophobic dye Bodipy® 493/503, which gives a green fluorescence and specifically stains neutral lipids (Gocze and Freeman, 1994). Therefore, we examined wild-type and P_II_ deficient mutant cells by fluorescence microscopy. A strong intracellular fluorescence signal could be detected in some wild type cells taken from early exponential phase of growth, as exemplarily shown in Figure 5A. The number of lipid bodies per cell was determined and is shown in Figure 5B. Cells of the P_II_ mutant have at least one or two lipid bodies (mean 1.6 lipid droplets per cell), whereas only few cells have lipid bodies in wild type (mean 0.39 lipid droplets per cell). Lipid droplets formed transiently in the early phase of growth and disappeared with increasing optical densities, possibly being converted to phospholipids. To gain further insights in this phenotype, total lipids were extracted from exponentially growing cultures and the phospholipid content was analyzed using thin layer chromatography. No significant difference in phospholipid content was apparent between wild type and the P_II_ mutant excluding the accumulation of phospholipids in the observed vesicles. Hence the extracts were subjected to thin layer chromatography using a system, which is able to resolve more hydrophobic lipids (Figure 5C). Staining with iodine vapor revealed spots occurring in both wild type and P_II_ mutant and an additional spot only present in the P_II_ mutant. These spots migrate similar to a triacylglycerol standard (composed of C12, C14 and C16 triacylglycerols) and sesame oil (a complex mixture of C16 and various C18 fatty acids containing triacylglycerols). Stained spots were scraped off, extracted and converted to fatty acid methyl esters for GC/MS analysis. The lower spot contained primarily palmitic and stearic acid and minor traces of pentadecaonic and heptadecanoic fatty acid. The upper spot present in the P_II_ mutant contained primarily palmitic and stearic fatty acids (with no pentadecaonic and heptadecanoic fatty acids present). No unsaturated C16 or C18 fatty acids could be detected in both spots.

## Discussion

Previous work has demonstrated that the P_II_ protein GlnB from *A. thaliana*, as well as bacterial GlnB proteins from *Azospirillum brasilense* and *E. coli* interact with BCCP (Rodrigues et al., 2014) and change the biosynthetic activity of ACCase (Feria Bourrellier et al., 2010; Gerhardt et al., 2015). Here, the interaction of BCCP with GlnB could be confirmed for unicellular cyanobacteria, and for the first time, an implication of P_II_ signaling on acetyl-CoA metabolism could be demonstrated.

The effect of *Synechococcus* P_II_ on the reconstituted *E. coli* ACCase activity qualitatively matches the results of protein-protein interaction determined for *Sy*P_II_-BCCP interaction. This implies that wild-type P_II_ proteins from cyanobacteria tune down ACCase activity by binding to the BCCP subunit of ACCase, whilst a negative charge of the T-loop at position 49 (phosphomimetric mutants S49E and S49D) impairs ACCase regulation. Residue R103 of P_II_ is directly involved in salt bridge contact to the gamma-phosphate of ATP (Fokina et al., 2010a). Consequently, R103 mutants of P_II_ are affected in ATP binding and the inability of the R103 variant to regulate ACCase matches the strict ATP dependence of P_II_-BCCP interaction. Binding of effector molecules by P_II_ tremendously alters the conformation of its T-loop (Fokina et al., 2010a; Truan et al., 2014; Zeth et al., 2014; Forchhammer and Lüddecke, 2016), suggesting that the ATP requirement for P_II_-BCCP complex formation is due to the ATP-induced T-loop conformation of P_II_. Occupation of all three ATP binding sites seem required in order to form a stable GlnB-BCCP complex. The complex is destabilized by 2-OG concentrations that are 4-fold higher than the affinity constant of the second binding site (Fokina et al., 2010b), suggesting that binding of 2-OG to the third binding site determines the stability of the complex. This implies that all three T-loops of GlnB, which communicate with the ligand binding sites, are involved in complex formation with BCCP. By contrast, using the reconstituted ACC from *E. coli* as assay system, GlnB mediated activity inhibition could be relieved low 2-OG concentrations (IC_50_ value of only 4.8 μM), which were well below the concentration required to inhibit formation of the BCCP-GlnB complex (42 μM). It is likely, that subtle conformational changes of the P_II_ T-loop in the GlnB-BCCP complex caused by 2-OG binding to the high affinity binding site 1 (K_*d*_ = 5.1 μM) cause this effect. A similar post-binding effect has been observed for the P_II_ target NtrB in *E. coli*, where P_II_ in complex with NtrB regulated the phosphatase activity in response to 2-OG, an effect that was termed post binding regulation (Jiang and Ninfa, 2009).

The importance of the T-loop for complex formation was clearly highlighted by the phosphomimetic variants of P_II_ where the negative charge at T-loop position 49 strongly impaired GlnB BCCP interaction. In case of the S49D variant, this could be partially overcome by applying excess ATP concentrations. Apparently, electrostatic repulsion hinders the T-loop to adopt the proper conformation for BCCP binding, which back couples to the ATP binding site. Interestingly, the charge neutral substitution S49C also had an effect on the interaction and required increased ATP concentrations (EC_50_ 143 μM) to enable GlnB BCCP interaction. This effect might be caused by sterical hindrance due to increased bulkiness of the T-loop and to compensate this distortion, increased ATP concentrations were required enforce the T–loop in the BCCP-accepting conformation. Interestingly the I86N variant, which is locked in a compact T-loop conformation (Fokina et al., 2010b) was completely unable to exhibit regulation on ACC activity, emphasizing that the T-loop conformation plays a critical role in ACC inhibition. Which specific T-loop conformation elicits inhibition of ACC remains to be elucidated from a structural biological perspective.

Gerhardt et al. (2015) demonstrated that the interaction of GlnB with ACCase tunes down the k_*cat*_ of the reaction 3.5 times but does not affect the K_*M*_ value of *E. coli* ACC toward acetyl-CoA, for which a K_*M*_ of 228 μM was determined. Assuming a cell volume of 0.5 μl for 1*10^8^ cells allows an estimation of the intracellular acetyl-CoA concentration in the wild type and the P_II_ mutant. At growth conditions, where a low phosphorylation status of P_II_ is expected, and consequently, P_II_ complexed to BCCP, the acetyl-CoA concentrations of the wild type were in the range of 226–310 μM, which is close to the K_*M*_ for ACC. When conditions change toward increased P_II_ phosphorylation, dissociation of the P_II_ BCCP complex is expected and hence, acceleration of ACCase activity. This should lead to an immediate draining of the acetyl-CoA pool below the K_M_ for ACCase. The turn-over of the reaction will necessarily slow down and the acetyl-CoA pool will finally reach a new equilibrium. This is in fact observed during nitrogen starvation, growth with nitrate and CO_2_, or the P_II_ mutant (52–74 μM). The total flux through the ACCase reaction is, however, not strongly affected in such a steady state. Solely the factor that limits the over-all reaction is different: either ACCase is limited by interaction with P_II_ (in presence of high acetyl-CoA levels) or by low acetyl-CoA levels (in the absence of P_II_ interaction). The regulatory impact of T-loop modification of P_II_ on ACCase control and acetyl-CoA levels was clearly revealed through complementation with P_II_ variants. The *in vivo* acetyl-CoA levels of the S49E complemented variant remained as low as in the P_II_ deficient mutant, but could be recovered by complementation with native P_II_.

In line with these kinetic considerations above, the fatty acid levels in the wild type and the P_II_ mutant were quite similar under most tested conditions and only significantly different when cells were grown with ammonia (${\text{HCO}}_{3}^{-}$ or air bubbling as carbon source). Steady-state malonyl-CoA levels are 10 times lower than acetyl-CoA levels (Bennett et al., 2009). This is in agreement with the ACCase reaction being the rate-limiting step in fatty acid synthesis, whereas the condensation reaction is efficiently consuming malonyl-CoA. Therefore, the activity regulation of ACCase by P_II_ is unlikely to affect the hardly detectable cellular malonyl-CoA levels. Fatty acids are primarily present in phospholipids, which build up the outer, cytoplasmic and thylakoid membranes. Due to the abundant membrane system present in cyanobacteria, the corresponding fatty acid pool is big and less prone to fluctuations. Acetyl-CoA on the contrary is quickly turned over and used in various anabolic reactions, while the pool size is comparably low (see above) and prone to fluctuations based on the carbon or nitrogen supply. Hence, a tight regulation of ACCase is necessary to control the size of this important metabolite pool, without strongly affecting the pool of fatty acids. Interestingly the fatty acid distribution was slightly shifted toward C16 fatty acids, which were more abundant in the P_II_ mutant and the S49E complemented strain.

The two main metabolic routes which provide the cell with acetyl-CoA are CO_2_ fixation through the Calvin-Benson-Bessham cycle (CBB) or degradation of glycogen through various pathways (Xiong et al., 2015; Chen et al., 2016). The biggest differences in acetyl-CoA pools were visible in the first 48 h of growth. Conversely, total fatty acid levels were slightly higher in the P_II_ mutant and the S49E complemented strain during this early period of growth. This growth period is characterized by degradation of internal carbon reserves to provide carbon and energy for growth. Furthermore, in the early growth phase, when the optical density of the culture is still low, photosynthetic activity is at its maximum. Since nitrogen is abundant in this growth phase, P_II_ should interact with ACC to keep the acetyl-CoA levels high, thereby slightly reducing the synthesis of fatty acids. The high acetyl-CoA levels could be beneficial for other anabolic reactions, which require acetyl-CoA, such as the synthesis of arginine (N-acetyl-glutamate) or leucine (synthesis of α-isopropylmalate). Furthermore, acetyl-CoA levels assure carbon flux into the citric acid cycle to maintain the GS-GOGAT cycle, which is constantly depleted through nitrogen assimilation. Moreover, high acetyl-CoA levels could play a role for protein acetylation, which was recently demonstrated to be abundant in *Synechocystis* (Mo et al., 2015), but it is so far unclear how acetylation influences the enzymatic activities of those enzymes.

Transition to the light-limited linear growth phase at higher optical densities correlated with reduced acetyl-CoA levels. In this phase of growth, light intensity decreases due to self-shading of the cells, which limits photosynthesis and slows down growth (Foster et al., 2007). This negatively affects CO_2_ fixation, and consequently, the acetyl-CoA pools, replenished by CO_2_ fixation products, decrease during the linear growth in the wild type and P_II_ complemented strain. As a consequence, the fatty acid levels became indistinguishable between wild-type the P_II_ deficient mutant.

The observation that throughout the growth phase, acetyl-CoA levels decreased has previously been reported also from *E. coli* (Chohnan and Takamura, 1991). These authors have argued that the carbon supply in form of glucose is key to high intracellular acetyl-CoA levels in *E. coli*. However, control by the P_II_ regulatory system might play an important role also in this case, an assumption, which requires further investigation. In contrast to the effect of P_II_ regulation in the early growth phase, other regulatory mechanisms so far known appear to inhibit ACC activity at later stages of growth (Jiang and Cronan, 1994; Meades et al., 2010).

Higher total fatty acid levels in the early exponential growth phase coincide with the transient appearance of lipid droplets, most prominently in the P_II_-deficient mutant. Lipid droplets are best known in eukaryotes and a recent report established a connection between lipid body formation and GlnB (Zalutskaya et al., 2015). Reduced levels of GlnB protein in the eukaryotic green algae *Chlamydomonas reinhardtii* increased the amount and the size of lipid bodies. Even though lipid bodies have been previously described in *Synechocystis* using electron microscopy, they were suggested to play a role in thylakoid maintenance (van de Meene et al., 2006). Within the last decade lipid droplets have emerged as intracellular inclusions also present in heterotrophic bacteria (Kalscheuer et al., 2001; Yang et al., 2012) or the filamentous cyanobacterium *Nostoc punctiforme* (Peramuna and Summers, 2014; Perez et al., 2016), where they contain triacylglycerides, α-tocopherol and alkanes (Peramuna and Summers, 2014). Isolated lipids of *Synechocystis* migrated on TLC similar to sesame oil and a triacylglycerol mixture and GC/MS analysis revealed that they primarily contained C16 and C18 saturated fatty acids with traces of pentadecanoic and heptadecanoic acid as has been observed in exponentially grown *N. punctiforme* (Peramuna and Summers, 2014). These lipids must therefore be triacylglycerols as TLC and GC/MS analysis suggest, even though diacylglycerol acyltransferase homologs are absent in the genome of *Synechocystis*. Lipid droplets disappeared in the later phases of growth and probably represent a dynamic reservoir for fatty acid storage (in form of TAG) and turnover (Yang et al., 2012). Although no triacylglycerol synthase has been identified in the genome of *Synechocystis*, the presence of a triacylglycerol lipase encoded by *sll1969* supports a functional triacyl-glycerol metabolism in this strain. This suggests a hitherto unknown triacylglycerol synthase in *Synechocystis* PCC 6803.

Taken together, this study showed that BCCP-GlnB interaction is present in the cyanobacterial linage and must have arose early in the evolution of P_II_ proteins, as it is present in distantly related bacterial lineages (Feria Bourrellier et al., 2010; Gerhardt et al., 2015). This regulation has later been transferred to the plant kingdom through cyanobacterial endosymbiosis, where it has been conserved in plant metabolism (Feria Bourrellier et al., 2010; Zalutskaya et al., 2015). The present study shows that interaction with BCCP allows P_II_ to control the cellular acetyl-CoA levels. P_II_ regulation of ACCase provides the opportunity for an intriguing regulatory feedback loop: low 2-OG levels promote P_II_-ACCase interaction and cause an increase in acetyl-CoA levels through restriction of ACCase activity. In turn, this could promote the flux into the oxidative branch of the TCA cycle, leading to increased 2-OG levels. Such a feedback loop could help in maintaining and balancing the 2-OG levels under nitrogen-rich conditions, but requires further investigation and experimental verification. Once carbon supply is limited, this is sensed by P_II_ through low 2-OG levels and according to our data, this enables the cell to limit fatty acid synthesis more efficiently than in the absence of P_II_ regulation. The fact that this interaction is conserved from bacteria to plants indicates a considerable selective advantage in fine-tuning metabolic homeostasis.

## Author contributions

EG: Performed and designed experiments with reconstituted *E. coli* ACC; KS: Performed and designed pull-down experiments, acetyl-CoA and total fatty acid quantifications; WH: Performed lipid analysis, designed and analyzed pull-down experiments, acetyl-CoA and total fatty acid quantifications; KF: Supervised the work and conceived and wrote the manuscript with LH and WH; All authors have read and approved the manuscript.

## Funding

This work was supported by DFG grant Fo195/9-2 and RTG1708. EG and LH acknowledge CNPq, INCT, CAPES and Fundação Araucária for the financial support.

### Conflict of interest statement

The authors declare that the research was conducted in the absence of any commercial or financial relationships that could be construed as a potential conflict of interest.
