# Supplementary figures and images for: Interaction of the Nitrogen Regulatory Protein GlnB (PII) with Biotin Carboxyl Carrier Protein (BCCP) Controls Acetyl-CoA Levels in the Cyanobacterium Synechocystis sp. PCC 6803
Source: Front Microbiol. 2016 Oct 26;7:1700. doi: 10.3389/fmicb.2016.01700 (PMC5080355; doi:10.3389/fmicb.2016.01700)

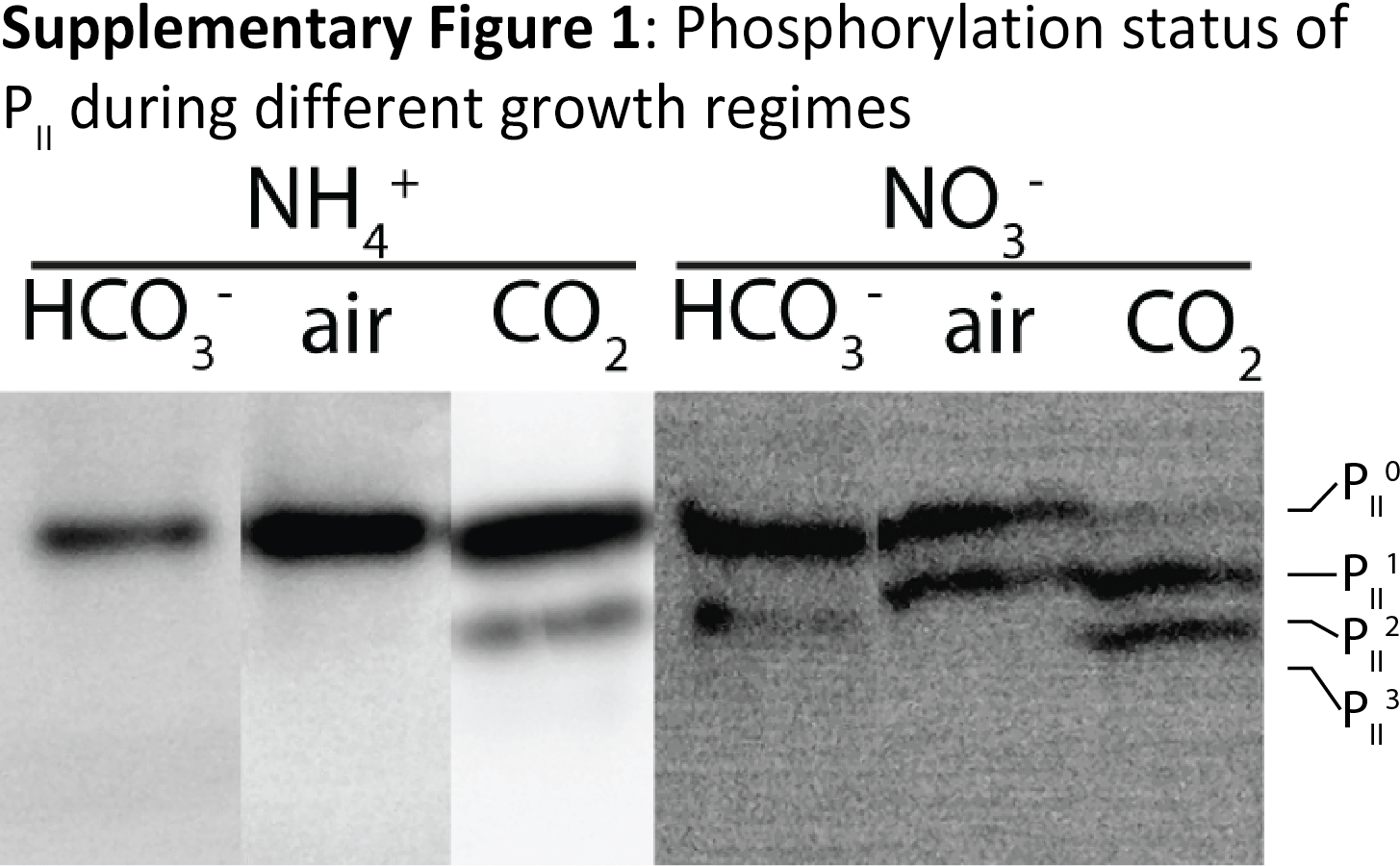

Supplement: Supplementary file 1 [file Image1.PNG]
